# ROCK signalling induced gene expression changes in mouse pancreatic ductal adenocarcinoma cells

**DOI:** 10.1038/sdata.2016.101

**Published:** 2016-11-08

**Authors:** Nicola Rath, Gabriela Kalna, William Clark, Michael F. Olson

**Affiliations:** 1 Cancer Research UK Beatson Institute, Garscube Estate, Switchback Road, Glasgow G61 1BD, UK; 2 Institute of Cancer Sciences, University of Glasgow, Glasgow G12 8QQ, UK

**Keywords:** Cancer, Cancer, RHO signalling, Actin, Stress fibres

## Abstract

The RhoA and RhoC GTPases act via the ROCK1 and ROCK2 kinases to promote actomyosin contraction, resulting in directly induced changes in cytoskeleton structures and altered gene transcription via several possible indirect routes. Elevated activation of the Rho/ROCK pathway has been reported in several diseases and pathological conditions, including disorders of the central nervous system, cardiovascular dysfunctions and cancer. To determine how increased ROCK signalling affected gene expression in pancreatic ductal adenocarcinoma (PDAC) cells, we transduced mouse PDAC cell lines with retroviral constructs encoding fusion proteins that enable conditional activation of ROCK1 or ROCK2, and subsequently performed RNA sequencing (RNA-Seq) using the Illumina NextSeq 500 platform. We describe how gene expression datasets were generated and validated by comparing data obtained by RNA-Seq with RT-qPCR results. Activation of ROCK1 or ROCK2 signalling induced significant changes in gene expression that could be used to determine how actomyosin contractility influences gene transcription in pancreatic cancer.

## Background & Summary

ROCK1 and ROCK2 are serine/threonine kinases that play key roles in actomyosin cytoskeleton dynamics^1^. Acting in response to activation of their upstream regulators RhoA and RhoC GTPases, their regulation of cytoskeletal architecture gives them central influence on cell morphology, adhesion and motility^2^. ROCK proteins have been discovered to have important roles in differentiation, proliferation and apoptosis/survival in various cell types^3^. Given their roles in regulating cell motility, there has been considerable interest in developing ROCK inhibitors as cancer therapeutics to target invasion and metastasis^4,5^. It has more recently become appreciated that Rho-mediated actomyosin dynamics also impact gene transcription^6^. Despite many years of research, the effect of ROCK kinases in normal cell function or in disease states on the regulation of gene expression has not been well characterized.

Pancreatic cancer is the fourth leading cause of cancer-related deaths in the western world^7,8^. The most common form is pancreatic ductal adenocarcinoma (PDAC), with the majority of patients carrying activating *KRAS* oncogene mutations and mutation or deletion of the *TP53* tumour suppressor^9^. Furthermore, exome sequencing of pancreatic cancer genomes revealed that ~15% of pancreatic cancer patients carry *ROCK1* gene amplifications^10^. Additional genetic alterations that reduce TGFβ signalling, for example through loss of SMAD4 expression, result in elevated ROCK1-dependent cell contractility that promotes PDAC progression^11,12^. As a result, there are clear indications that ROCK signalling contributes to PDAC growth and progression, likely as an ancillary factor, through mechanisms that remain to be determined.

**Figure 1:**
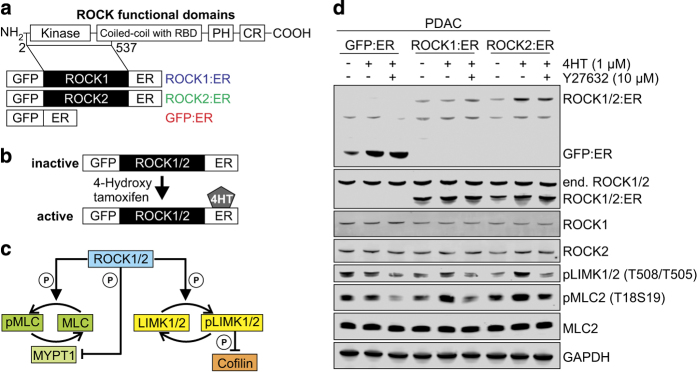
ROCK kinase activation in PDAC cells. (**a**) ROCK functional domains and ROCK kinase expression constructs (CR, cysteine-rich; ER, estrogen receptor hormone binding domain; GFP, green fluorescent protein; PH, pleckstrin homology domain; RBD, Rho binding domain). (**b**) Conditional activation of ROCK kinase by 4-hydroxytamoxifen (4HT). (**c**) Overview of the ROCK signalling pathway. (**d**) PDAC cells expressing GFP:ER, ROCK1:ER or ROCK2:ER fusion proteins were treated with EtOH vehicle or 1 μM 4HT in the presence or absence of 10 μM Y27632 ROCK inhibitor. Immunoblotting shows ER-fusion proteins, endogenous ROCK1 and ROCK2, and phosphorylation status of ROCK targets LIMK1/2 (T508/T505) and regulatory myosin light chain (MLC2; T18S19). Total MLC2 and glyceraldehyde-3-phosphate dehydrogenase (GAPDH) were blotted as loading controls.

To investigate the contribution elevated ROCK signalling has on pancreatic cancer progression, we used pancreatic cancer cells previously isolated from a mouse PDAC tumour driven by *Kras*^*G12D*^*/p53*^*+/*−^ (ref. 13) and retrovirally transduced constructs encoding ROCK1:ER, ROCK2:ER or GFP:ER fusion proteins (Fig. 1a)^14^. The stable fusion protein expressing cell lines allow for conditional activation of ROCK kinase signalling by the addition of estrogen analogues including 4-hydroxytamoxifen (4HT) (Fig. 1b), which induces actomyosin contraction through phosphorylation and inhibition of the myosin phosphatase targeting subunit (MYPT) 1 and direct phosphorylation of the myosin light chains (MLC) (Fig. 1c). This is aided by LIM kinase 1 and 2 (LIMK1/2) phosphorylation, which in turn phosphorylate and inactivate the actin-severing cofilin proteins leading to actin filament stabilisation (Fig. 1c). Western blotting showed expression of GFP:ER, ROCK1:ER or ROCK2:ER fusion proteins in PDAC cells, with ROCK1:ER or ROCK2:ER expression comparable to endogenous ROCK1 or ROCK2 (Fig. 1d). To demonstrate conditional ROCK:ER activation, immunoblotting revealed 4HT-induced activation of ROCK1:ER or ROCK2:ER leading to increased MLC and LIMK1/2 phosphorylation that could be reversed by the inclusion of the ROCK inhibitor Y27632 (Fig. 1d).

**Figure 2:**
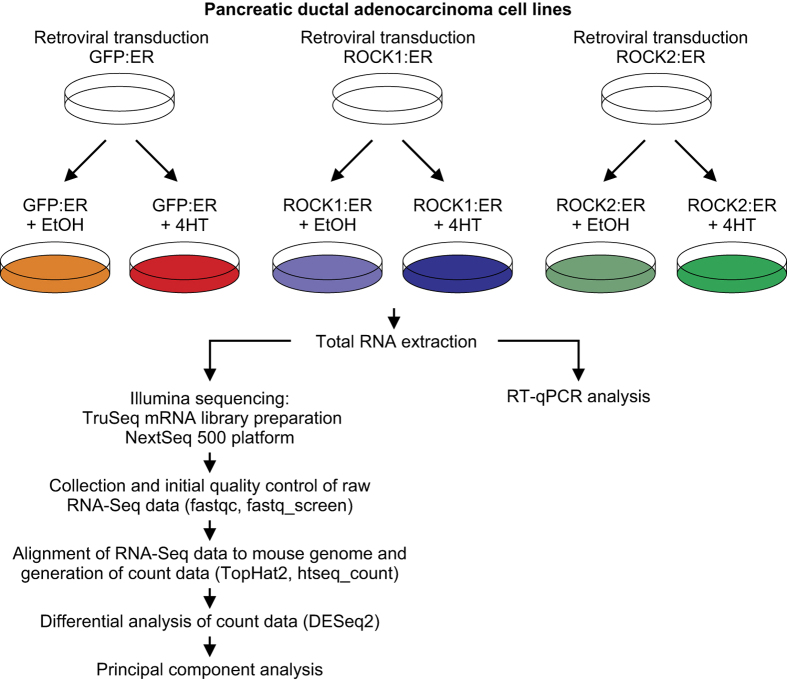
Overview and experimental design of the study.

To study the global effect of ROCK activation on gene expression in mouse PDAC cells, we sequenced polyA+ mRNAs of GFP:ER expressing cells treated with EtOH vehicle or 4HT, ROCK1:ER expressing cells treated with 4HT and ROCK2:ER cells treated with 4HT from 3 independent experiments (Fig. 2,Table 1). The study has been described at the NCBI Bioproject (Data Citation 1), with a description of the cells used at the NCBI BioSample (Data Citation 2). Primary data are available at the NCBI Sequence Read Archive (Data Citation 3).

## Methods

### Cell culture

A pancreatic ductal adenocarcinoma (PDAC) cell line was generated from the pancreatic tumour of a LSL-Kras^G12D/+^; LSL-p53^fl/+^; Pdx1-Cre mouse^13^. Cells were maintained in High glucose DMEM (Gibco 21969-035) containing 10% fetal bovine serum (FBS; Gibco), 2 mmol l^−1^ L-glutamine (Gibco), and penicillin-streptomycin (Gibco).

### Generation of stable cell lines

Retroviral infection was used to generate stable PDAC cell lines expressing conditionally active human ROCK1 (ROCK1:ER), human ROCK2 (ROCK2:ER) or GFP control (GFP:ER). The retroviral pBABE puro constructs have been described previously^14,15^. Stable PDAC cell pools were selected using 2.5 μg ml^−1^ Puromycin using standard procedures.

### Western blot analysis

5×10^5^ cells were seeded into 6-well plates in DMEM and allowed to settle and grow overnight. Next day, cells were starved in serum-free DMEM for 9 h, followed by serum-free DMEM with EtOH vehicle or 1 μM 4HT (Sigma H7904) in the presence or absence of 10 μM Y27632 (Tocris 1254). After 16 h of treatment, whole cell lysates were prepared in cell lysis buffer (1% SDS, 50 mM Tris pH 7.5) and protein concentration determined by Bicinchoninic assay (Sigma). Standard protocols were used for Western blotting. Signals were detected by infrared imaging (Li-Cor Odyssey). Antibodies used: ROCK1 (BD-611136), ROCK2 (BD-610623), ROCK1/2 (Millipore 07-1458), phospho-Limk1/2 (Cell Signalling 3841), phospho-MLC2 (Cell Signalling 3674), MLC2 (Cell Signalling 3672), GFP (Abcam ab6556), GAPDH (Millipore MAB374), Alexa-Fluor 680 (life technologies) and DyLight 800 (Thermo Fisher Scientific).

### RNA isolation

1×10^6^ cells were seeded into 6-well plates in DMEM and allowed to settle and grow overnight. Next day, cells were washed three times with serum-free DMEM, then serum-free DMEM with EtOH vehicle or 1 μM 4HT (Sigma H7904) was added to the cells for 24 h. Cells were harvested with Trypsin and total RNA was extracted using the RNAeasy kit (Qiagen) according to manufacturer’s instructions. RNA was quantified using the Nanodrop spectrophotometer (Nanodrop Tech). The Agilent RNA ScreenTape assay and the Agilent 2200 TapeStation system were used to determine the RNA integrity number equivalent (RINe).

### RNA sequencing

Total RNA was used to generate an oligo dT-enriched library with the Illumina TruSeq RNA Library Preparation kit v2.0. RNA fragmentation yielded fragments for use in preparation of the DNA library ranging from 120 to 210 nucleotides with a median of 155 nucleotides. Quality and quantity of the DNA library was assessed using the Agilent 2100 Bioanalyzer and the Qubit (Thermo Fisher Scientific), respectively. The library was run on the Illumina NextSeq 500 platform using the High Output 75 cycles kit (2×36 cycles, paired-end reads, single index).

### RNA sequence analysis

Quality control checks of raw RNA-Seq data files were done with fastqc (http://www.bioinformatics.babraham.ac.uk/projects/fastqc/) and fastq_screen (http://www.bioinformatics.babraham.ac.uk/projects/fastq_screen/). RNA-Seq reads were aligned to the mouse genome (GRCm38.75) using TopHat2.0.10 (ref. 16) and BAM files were further processed with HTseq0.5.4p3 (http://www.huber.embl.de/users/anders/HTSeq/doc/count.html) using standard configurations. The percentage of aligned reads was 88.6%±2.0% (mean±s.d.). Differential analysis of count data was performed by the DESeq2 package (DESeq2)^17^. Regularized log transformation was used to transform the DESeq2 data for principal component analysis.

### Quantitative PCR

Total RNA was used to synthesize complementary DNA with the QuantiTect Reverse Transcription Kit (Qiagen). Quantitative polymerase chain reactions (qPCR) were set up with the DyNAmo HS SYBR Green qPCR Kit (Thermo Fisher Scientific) and primers for *Ptgs2*, *Tff3*, *Tnc*, *Cd44*, *Cyr61* or *Gapdh* (Quantitect Primer Assay, Qiagen). Reactions were run and analysed using the 7500 Fast Real-Time PCR System (Applied Biosystems).

## Data Records

Unprocessed RNA sequencing reads have been deposited as fastq files at the National Center for Biotechnology Information (NCBI) Sequence Reads Archive (SRA) with the reference SRP081135. In addition, a project overview has been submitted as the BioProject reference PRJNA327913 (http://www.ncbi.nlm.nih.gov/bioproject/) (Data Citation 1) with a description of the BioSample reference SAMN05361890 (http://www.ncbi.nlm.nih.gov/biosample/) (Data Citation 2).

The fastq files correspond to three independent experimental replicates (Experiment numbers 1–3) for the PDAC expressing GFP:ER cells treated with EtOH vehicle control or 4HT, or for the ROCK1:ER or ROCK2:ER expressing cells treated with 4HT as indicated in Table 1. Forward (R1) and reverse (R2) reads have been combined, with SRA accession numbers for the combined sequencing results also indicated in Table 1 (Data Citation 3). Please also see the associated Metadata Record.

## Technical Validation

### Quality control of RNA-Seq data

**Figure 3:**
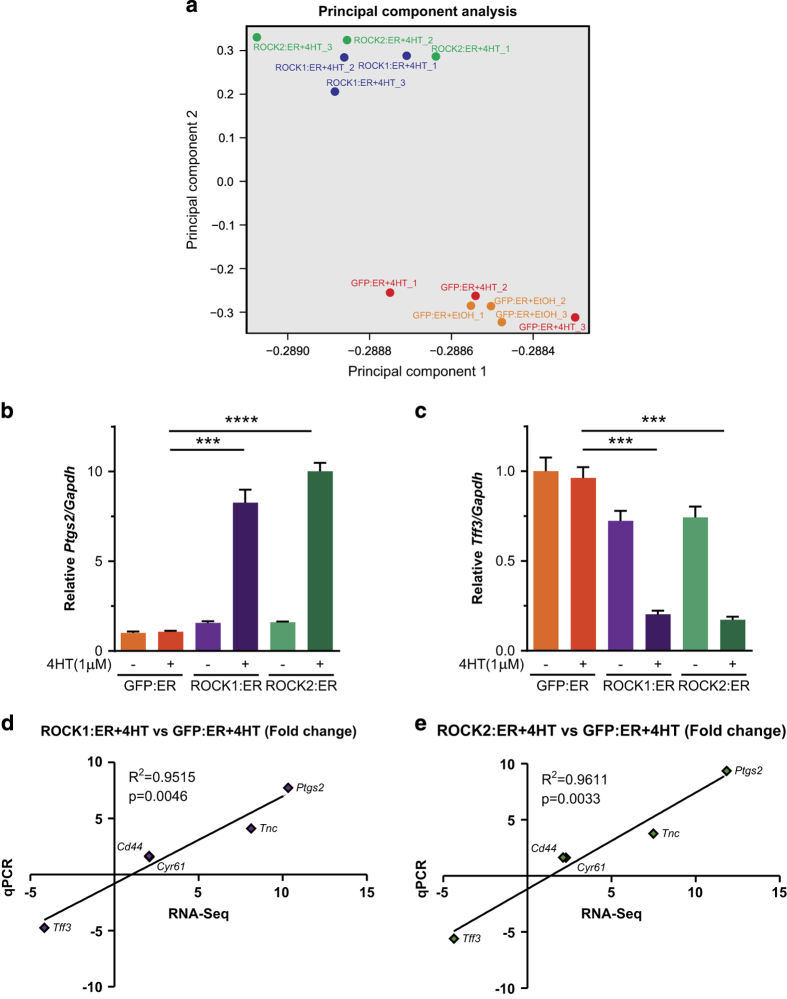
Quality control of RNA-Seq data. (**a**) Principal component plot of RNA-Seq (DESeq2) data indicating the clustering together of GFP:ER samples treated with vehicle control or 4HT, and ROCK1:ER plus ROCK2:ER samples treated with 4HT. (**b**) PDAC cells expressing GFP:ER, ROCK1:ER or (**c**) ROCK2:ER were treated with EtOH vehicle or 1 μM 4HT. *Ptgs2* and *Tff3* mRNA levels relative to *Gapdh* housekeeping gene were determined by qPCR. Means±s.e.m., unpaired *t*-test (*n*=3). ***P<0.001. (**d**) Linear regression plots of fold change for GFP:ER+4HT versus ROCK1:ER+4HT or (**e**) GFP:ER+4HT versus ROCK2:ER+4HT obtained by RNA-Seq (DESeq2 data) compared to RT-qPCR (relative to *Gapdh*). RNA transcripts up-regulated by ROCK activation: *Ptgs2*, *Tnc*, *Cd44* and *Cyr61*. RNA transcript down-regulated by ROCK activation: *Tff3*. R^2^ of Goodness of Fit with p value (*n*=3).

RNA quality was confirmed using the Agilent RNA ScreenTape assay and the Agilent 2200 TapeStation system, which revealed RNA integrity number equivalent (RIN^e^) values of 10 for all samples. Following RNA-seq, principal component analysis indicated that GFP:ER samples that had been treated with EtOH vehicle or 4HT clustered together while ROCK1:ER and ROCK2:ER samples treated with 4HT clustered together separate from the GFP:ER grouping (Fig. 3a), consistent with the conditional activation of ROCK catalytic activity observed by western blotting (Fig. 1d). Quantitative reverse transcription PCR (RT-qPCR) analyses validated differences in gene expression upon ROCK1:ER or ROCK2:ER activation identified by RNA seq, including increased Protaglandin-endoperoxidase 2 (*Ptgs2*) RNA (Fig. 3b) and decreased Trefoil factor 3 (*Tff3*) RNA (Figure 3c). We further validated the changes in gene expression identified by RNA seq by comparing fold changes in sequence reads with fold changes determined by RT-qPCR for *Ptgs2*, *Tff3*, *Cyr61*, *Tnc* and *Cd44* between 4HT treated GFP:ER versus ROCK1:ER (Fig. 3d) and GFP:ER versus ROCK2:ER (Fig. 3e) conditions. In both cases, the fold-changes determined by either method fell on a single fitted straight line with R^2^>0.95 and *P*<0.05 (Fig. 3d,e). Although there was good agreement between RNA seq and RT-qPCR for this limited gene set, it is possible that the analytical methods used may have underestimated gene expression levels^18^.

## Additional Information

**How to cite**: Rath, N. *et al.* ROCK signalling induced gene expression changes in mouse pancreatic ductal adenocarcinoma cells. *Sci. Data* 3:160101 doi: 10.1038/sdata.2016.101 (2016).

**Publisher’s note**: Springer Nature remains neutral with regard to jurisdictional claims in published maps and institutional affiliations.

## Supplementary Material



## Figures and Tables

**Table 1:** Description and names of data files deposited with SRA.

**Experiment**	**Fusion protein**	**Treatment**	**Data File ID**	**Total sequence reads**	**SRA accession**
1	GFP:ER	Ethanol (vehicle)	1-EX1-GFP-EtOH	3.8E+07	SRR4015461
1	GFP:ER	4HT (1 μM)	2-EX1-GFP-4HT	6.4E+07	SRR4015530
1	ROCK1:ER	4HT (1 μM)	3-EX1-ROCK1–4HT	5.8E+07	SRR4015566
1	ROCK2:ER	4HT (1 μM)	4-EX1-ROCK2-4HT	6.2E+07	SRR4015572
2	GFP:ER	Ethanol (vehicle)	5-EX2-GFP-EtOH	6.8E+07	SRR4014833
2	GFP:ER	4HT (1 μM)	6-EX2-GFP-4HT	6.3E+07	SRR4015468
2	ROCK1:ER	4HT (1 μM)	7-EX2-ROCK1-4HT	5.7E+07	SRR4015485
2	ROCK2:ER	4HT (1 μM)	8-EX2-ROCK2-4HT	7E+07	SRR4015492
3	GFP:ER	Ethanol (vehicle)	9-EX3-GFP-EtOH	6.1E+07	SRR4015510
3	GFP:ER	4HT (1 μM)	10-EX3-GFP-4HT	6.3E+07	SRR4015519
3	ROCK1:ER	4HT (1 μM)	11-EX3-ROCK1-4HT	6.1E+07	SRR4015548
3	ROCK2:ER	4HT (1 μM)	12-EX3-ROCK2-4HT	6.3E+07	SRR4015560
